# Factors Associated with Male Partner Involvement in Programs for the Prevention of Mother-to-Child Transmission of HIV in Rural South Africa

**DOI:** 10.3390/ijerph14111333

**Published:** 2017-11-01

**Authors:** Motlagabo G. Matseke, Robert A. C. Ruiter, Violeta J. Rodriguez, Karl Peltzer, Geoffrey Setswe, Sibusiso Sifunda

**Affiliations:** 1 Human Sciences Research Council, Pretoria 0001, South Africa; kpeltzer@hsrc.ac.za (K.P.); ssifunda@hsrc.ac.za (S.S.); 2 Department of Work and Social Psychology, Maastricht University, 6200 MD Maastricht, The Netherlands; r.ruiter@maastrichtuniversity.nl; 3 Department of Psychiatry & Behavioral Sciences, University of Miami Miller School of Medicine, Miami, FL 33136, USA; vjr5@med.miami.edu; 4 Department of Psychology, University of Georgia, Athens, GA 30602, USA; 5 Department of Public Health, University of Venda, Thohoyandou 0950, South Africa; gsetswe5@hotmail.com

**Keywords:** prevention of mother-to-child transmission of HIV (PMTCT), male partner involvement (MPI), pregnant women, rural South Africa

## Abstract

Male partner involvement (MPI) can contribute to the success of programs aimed at preventing mother-to-child transmission (PMTCT) of HIV. However, the definition and measures of MPI differ according to context. This study utilized secondary cross-sectional data to investigate the prevalence and determinants of MPI among 463 male partners of HIV-infected pregnant women in rural South Africa. Results indicated that 44.1% of male partners reported involvement in most or all specified male partner involvement activities (i.e., scores of 7 to 9). Descriptive, correlation and multiple linear-regression analyses were conducted. Positive predictors of MPI included relationship status, own HIV status, awareness of female partner’s positive HIV status, female partner’s desire to have more children, having family planning discussions with provider, condom use to prevent HIV and sexually transmitted infections (STIs), and partner reasoning skills. Negative predictors included partner verbal aggression. Overall, although MPI is low, the study underlines important information that could be used to develop interventions aimed at improving maternal and infant health in PMTCT programs in South Africa.

## 1. Introduction

There is ample evidence documenting the positive contributions men can make in the successful prevention of mother-to-child transmission of HIV (PMTCT) [1]. As such, male partner involvement (MPI) in PMTCT programs has been promoted as one of the priority interventions to improve PMTCT outcomes in sub-Saharan African countries [1,2]. In South Africa, the National Strategic Plan on HIV, STIs and tuberculosis (TB) promotes the involvement and engagement of men to strengthen PMTCT programs [3]. However, the realization of MPI in sub-Saharan countries is challenging because of male-related and structural factors [4].

In many countries, sexual and reproductive health (SRH) programs and services are centered around women, with men often lacking information that could assist them in making decisions regarding healthy behaviors and the roles they could play to promote overall family health, including accessing HIV prevention, care and treatment services [1]. Men’s constructive involvement has led to positive health outcomes for women, children and families [5]. Studies have demonstrated that men are interested in being involved in health promotion for their families and communities, such as in PMTCT and family-planning programs [1,5,6].

Male partner involvement in PMTCT counters prevailing traditional gender norms in many patriarchal societies in sub-Saharan Africa [1]. Men have seen health seeking in SRH to be a “women’s task”. They have generally seen the antenatal clinic as a space meant for women, and the definition and organization of the antenatal care program as fundamentally female-oriented [1,7]. Predictably, men think that antenatal clinic activities fall outside their area of responsibility [8,9]. They perceive that visiting the antenatal clinic would be “unmanly” [1,10,11], and therefore feel uncomfortable at the thought of being the only man present in the antenatal clinic [1,9] and fear stigmatization by other men [7,9,12].

Currently, there are no standardized measures of MPI, and its definition, meaning and the way it is understood may vary in different contexts. The most-common indicator used to define MPI is “men attending antenatal care appointments with their pregnant partner” [10,13,14]. Furthermore, MPI is also defined as men taking part in their pregnant partner’s birth plans, encouraging exclusive breastfeeding and immunization for their children, providing support during pregnancy and communicating with their partner about pregnancy-related health care [14,15]. It is, however, important to note that MPI cannot be comprehensively measured by a single indicator, hence some studies have used multiple indicators to form an index that captures a broader notion of MPI [12,16,17]. These more-sophisticated and composite measures of MPI would thus include indicators that capture different aspects of maternal and infant health, such as “accompaniment to antenatal care appointments”, “financial support” and “couple communication”. For example, Byamugisha et al. [12] used an MPI index using six indicators, namely, the “male partner accompanying his wife to antenatal care appointment”, “knowing the antenatal care schedule”, “discussing the antenatal care activities with female partner”, “providing money for antenatal care fees”, “knowledge of what happens at the antenatal care visit” and “condom use with the female partner during the current pregnancy”.

Psychosocial and socio-demographic factors, as well as health facility-related factors, have been associated with MPI in maternal and infant health services in sub-Saharan Africa [18]. Male partner involvement is determined by psychosocial factors such as individual beliefs and attitudes, intra-spousal or partner communication, and health facility-related factors such as opening hours of services, staff attitudes and behavior, and the non-male-friendly space [18]. Furthermore, socio-demographic characteristics, such as age, level of education, relationship status and employment status were found to be significantly associated with MPI in PMTCT [12,17,18,19]. Older age, higher educational attainment, being employed, cohabiting and monogamous marriages were found to be positively associated with MPI [18,19,20] as well as men’s knowledge in maternal and infant health and the number of children [17,21]. Other factors associated with men’s involvement include awareness of personal serostatus and having heard about PMTCT [12]. Men who had heard about PMTCT were two times more likely to become involved than those who had not. Male partners’ reports of having previously discussed HIV testing with female partners was significantly associated with their attendance at the antenatal clinic [12], whereas alcohol use by male partners and poor intra-spousal or partner communication was associated with poor MPI [18].

In addition to the above-mentioned factors identified in the empirical literature, the social-support concept was used as a supporting theoretical framework to explain MPI in this study. Social support may have a positive influence on health, and is described as the help provided through social relationships and interactions [22]. Four types of social support are identified, that is, emotional support (provision of empathy, love, trust and caring), instrumental support (provision of tangible aid and services), informational support (advice, suggestions and information) and appraisal support (provision of feedback useful for self-reevaluation and affirmation). For example, a male partner could show support to a pregnant partner by providing tangible aid and services such as accompanying her to the antenatal care appointment and reducing household workload during pregnancy (instrumental and emotional support). Many other types of support are possible.

Determinants of MPI in maternal and infant health and PMTCT have been studied in some countries [17,23,24]. There is, however, a lack of information regarding factors associated with MPI in maternal and infant health, and PMTCT among South African men. The aim of this study is to utilize secondary cross-sectional data to report on the prevalence of MPI and its determinants among male partners of HIV-infected pregnant women visiting primary health care facilities in rural South Africa.

## 2. Methods

### 2.1. Study Setting

This study utilized cross-sectional baseline data collected through the “Protect Your Family” (PYF) clinic-randomized controlled trial aimed at increasing the adherence and uptake of PMTCT protocols and increasing male partner involvement in antenatal and postnatal care. The study was conducted in 12 community health centers (CHCs) based in Gert Sibande and Nkangala districts in Mpumalanga province, South Africa [25]. The PYF trial protocol has been previously published [25] and is registered on clinicaltrials.gov, number NCT02085356.

Baseline data was collected among pregnant women and their male partners during the recruitment phase of the clinic-randomized trial from April 2015 to January 2017.

### 2.2. Participants and Procedures

Participants were 463 male partners who were recruited by virtue of being the partner of the HIV-infected pregnant woman and were systematically sampled (every consecutive patient after HIV post-test counselling). The “Protect Your Family” clinic-randomized controlled trial aimed to recruit 720 HIV-infected women together with their male partners. Of the 715 HIV-infected women who participated in the study, 174 of the male partners declined, and 78 had incomplete data resulting in a total sample of 463 male participants. To be eligible for participation in the study, women had to be six months or less pregnant, 18 years and older, HIV seropositive and have a male partner. Interested participants were given an appointment, and enrolled into the study after provision of informed consent. Once enrolled, all participants completed an assessment in their preferred language (English, isiZulu, or Sesotho) to enhance disclosure and accommodate different levels of literacy. Assessors were available at all times and always completed the demographic component of the assessment with the participant, to familiarize them with the software and enhance privacy during completion of the remainder of the assessment.

### 2.3. Measures

The data-collection instrument was developed through the Questionnaire Development System’s (QDS) Audio Computer-Assisted Self-Interview (ACASI) software. It included sections regarding demographic information, condom use, intimate partner violence, externalized HIV stigma, MPI, HIV knowledge, as well as additional sections such as HIV medication adherence and family-planning assessment.

#### 2.3.1. Socio-Demographic Information

Questions were asked to assess age, language, religion, level of education, employment status, relationship status, income and number of children (see Table 1) of the participants.

#### 2.3.2. Male Partner Involvement (MPI)

The main outcome variable of interest was male partner involvement, which was assessed using the MPI index adapted from a previous similar study [12]. The index comprised of 10 items related to the participants’ participation in specific areas of antenatal care, including PMTCT. However, for the purposes of this paper, a nine-item scale was used. One item was omitted from the scale due to the fact that it had too many missing responses, which negatively affected the reliability of the scale. Some of the nine questions included were, “Do you attend antenatal care visits with your pregnant partner?”, “Do you know your partner’s antenatal appointment times?”, “Have you discussed antenatal health care for your baby with your partner?”, “Do you support your partner’s antenatal visits financially?” and “Do you know what happens in the antenatal clinic?” Participants responded to each item as 1 “yes” or 0 “no”. All items were summed up to form a scale or composite MPI variable, (scores range 0–9; Cronbach’s alpha = 0.84), with higher scores indicating higher levels of MPI.

#### 2.3.3. HIV and PMTCT Knowledge

HIV and PMTCT knowledge was assessed using an adapted version of the AIDS-Related Knowledge Scale consisting of 18 items adapted from the Brief HIV PMTCT Knowledge questionnaire [26,27]. The first twelve items were questions or statements concerning modes of HIV transmission (e.g., Can a person get the AIDS virus through someone sneezing, like a cold or flu?), reinfection with resistant virus (e.g., If both partners are HIV positive it is okay to have unprotected sex) and condom use. The last six items were related specifically to PMTCT knowledge (e.g., Can an HIV positive mother infect her baby with HIV during pregnancy?). Responses to the scale items were categorized as “yes”, “no” and “don’t know”, or “true”, “false” and “don’t know”. All correct responses were scored as 1 while incorrect and “don’t know” responses were scored as 0. The scores were summed to form two indexes, that is, HIV knowledge (score range 0–12; Cronbach’s alpha = 0.78), and PMTCT knowledge (score range 0–6; Cronbach’s alpha = 0.72).

#### 2.3.4. Behavioral Characteristics

HIV testing was assessed by asking two questions, “Have you been tested for HIV?”, and “Are you HIV positive?” with either “yes” or “no” as responses to both questions. Condom use was assessed by asking the question “The last time you had sex, did you use a condom?” (“yes” or “no”). Disclosure of HIV status was assessed by using an adapted version of the Disclosure scale [28] which assesses disclosure among sexual partners and family members during pregnancy. Disclosure among sexual partners was considered for the purposes of this paper. Question asked was “is your spouse HIV positive?” with “0 = no, 1 = yes and 2 = I don’t know” as response categories. The preceding question asked was “Has your partner tested for HIV?” and the response categories were “0 = no, 1 = yes and 2 = I don’t know”. Total adherence to antiretroviral (ARV) medication was assessed by asking participants for each of the past seven days, “How much of your HIV medication did you take?” Response options were “none of my medication”, “half of my medication“, “all of my medication“. Self-rated adherence to ARV medication in the past four weeks was assessed by asking participants to rate their ability to take all their ARV medication as prescribed by the clinic staff. Response options were from 1, ”very poor”, to 6, “excellent“.

#### 2.3.5. Intimate Partner Violence (IPV)

IPV was assessed using an adapted version of the Conflict Tactics Scale 18 (CTS-18) [29]), which assesses reasoning, verbal aggression and mild and severe physical aggression through the use of different subscales. Respondents indicated the number of times in the past month their partner had engaged in specific behaviors using a scale of 0 (never) to 6 (more than 20 times). Scores on the summed scale ranged from 0 to 18, with higher scores indicating male partners experienced more violence. Cronbach’s alpha for the full scale in this sample was 0.86.

Scores on the reasoning sub-scale ranged from 0 to 18, with higher scores indicating more use of reasoning skills by female partners. Higher scores on the verbal aggression sub-scale (range 0–42) indicated more use of verbal aggression by female partners. Higher scores on the physical aggression sub-scale (range 0–48) showed more use of physical aggression by female partners. Cronbach’s alphas for the different subscales as used in the analysis are as follows: 0.73 for reasoning, 0.83 for verbal aggression, 0.91 for physical aggression (both mild and severe).

#### 2.3.6. AIDS-Related Stigma

The AIDS-Related Stigma Scale [30] is a nine-item subscale that measures externalized or public HIV stigma, for example, statements such as “People who have AIDS should be ashamed”, which are rated dichotomously using either a score of 0 (disagree) or 1 (agree). Scores on this scale ranged from 0 to 9, where higher scores indicate greater levels of stigma. Cronbach’s alpha in this sample was 0.67.

#### 2.3.7. Family-Planning Assessment

Family planning was assessed by asking questions on family-planning attitudes and contraception practices. Questions and items regarding contraception practices assessed whether the pregnancy was planned or unplanned, whether a provider was consulted prior to pregnancy, current use of family planning and intentions to engage in family planning in the future. Overall, seven questions and/or items were used for the purposes of this paper, that is, (1) “Are you the biological father of your partner’s baby?”, (2) “Was your partner’s pregnancy unplanned?”, (3) “Are you planning to have more children in the future?”, (4) “Are you currently using condoms to prevent HIV or sexually transmitted infections (STIs), how important the following things are when deciding to have a baby”, (5) “Your family’s desire for you to have a baby (or not)?”, (6) “Your partner’s desire to have a baby (or not)?”, (7) “Your doctor or health-care provider’s advice about whether you should have a baby or not?”. Responses to the questions were either “yes”, “no” or “not sure”, and for the items, responses were categorized as 1 through to 10, 1 = “not important at all” and 10 = “very important”.

### 2.4. Data Analysis

Statistical Package for Social Sciences (SPSS version 24.0, IBM, Armonk, NY, USA) was used to analyze the data. Frequency analyses were conducted to describe the socio-demographic and behavioral characteristics of the participants. To identify associations among the key study variables, Spearman or Pearson bivariate correlations were calculated; point-biserial correlations were used for dichotomous or categorical variables and phi coefficients for correlations between binary variables. Study variables that showed significant (*p* < 0.05) univariate associations with male partner involvement were included in a subsequent multiple linear-regression model to assess the amount of variance explained in male partner involvement. Then, to develop a final model, backward elimination was used to reduce the number of variables in the multiple linear-regression model; variables not significant at *p* < 0.10 were ultimately excluded. A number of observations (*n* = 7) with outlying model residuals were excluded from the final models, repeating the analyses until no more outlying residuals were identified.

### 2.5. Ethical Considerations

Prior to study onset, approval was obtained from the Human Sciences Research Council (HSRC) Research Ethics Committee (protocol number REC 4/21/08/13), the University of Miami Institutional Review Board (Human Subjects Research Office, HSRO study number 20130238), and the Mpumalanga Department of Health and Welfare (provincial, district, sub-district and clinic levels). Written informed consent for participation was obtained from each participant.

## 3. Results

### 3.1. Socio-Demographic and Behavioral Characteristics

The socio-demographic and behavioral characteristics of the sample as well as partner characteristics are shown in Table 1. The mean age of the 463 male partners was 33.04 years (SD = 7.24). Most of the male partners were unmarried and not living together (44.7%), were in a relationship for more than a year (82.5%) and had two or more children (48.2%).

Most (70.4%) reported to have tested for HIV, with 64.1% reporting to be HIV negative. About 75.2% reported to having not disclosed their status to their partner. PMTCT programs include routine HIV testing of women, however, only 81.6% reported that their partner had tested for HIV, with 67.2% reporting that their partner was HIV positive. The majority (58.8%) of those who reported that their partner was HIV positive also reported that their partner was on antiretroviral treatment. Just over half (52.5%) reported condom use at last sex, having more than three drinks of alcohol in the past month (40.4%), and most reported low levels of HIV stigma (median = 1.00, IQR = 0.00).

HIV knowledge and PMTCT-specific knowledge among male participants were generally high. Overall, respondents had a median HIV knowledge score of 10.00 (IQR = 3.00) from 12 items. With regards to PMTCT-specific knowledge, male partners had a median score of 5.00 (IQR = 3.00) from the six items. On average, participants showed low levels of verbal reasoning (median = 4.00, IQR = 7.00), verbal aggression (median = 1.00, IQR = 5.00) and physical aggression (median = 0.00, IQR = 0.00).

With regards to family planning assessment among the male participants, the majority (93.2%) reported being the biological father of the unborn baby in the current pregnancy. Seventy percent reported that they were currently using condoms to prevent HIV or sexually transmitted infections (STIs). Over half (50.1%) reported that they were planning to have more children in the future, and 27% reported having talked with a health-care provider about trying to get pregnant. With regards to making a decision to have a baby, most participants felt that their partner’s desire to have a baby was more important (median = 9.50, IQR = 9.00) than their health-care provider’s advice about whether to have a baby or not (median = 1.00, IQR = 9.00).

### 3.2. Level of Male Partner Involvement among Male Partners

Table 2 shows the prevalence of MPI across the different activities and for the summed activities. Over two thirds (63.7%) of the sample reported that they know their partner’s antenatal appointment time, while just over a third (31.5%) reported that they visited the antenatal clinic with their partner. The majority of the male partners (84.7%) reported that they financially supported their partner’s antenatal appointments. Almost half (49.7%) reported that they know what happens in the antenatal clinic, and 66.5% reported having discussed antenatal health care for their baby with their partner. Overall, participants obtained a median score of 6.00 (IQR = 4.00) for involvement in PMTCT. Only 10.6% reported to have participated in all specified activities (i.e., a highest MPI index score of 9).

### 3.3. Correlations with MPI

Correlations were computed between MPI and thirty-one (31) possible associated factors (see Supplementary Materials). The results show that correlations were statistically significant for only seventeen (17) factors. A small effect is *r* = 0.10–0.23, a moderate effect is *r* = 0.24–0.36 and a large effect is *r* ≥ 0.37 [31].

#### 3.3.1. Socio-Demographic Characteristics

Small positive associations with MPI were found for relationship status (specifically living together with partner) and number of children.

#### 3.3.2. Behavioral Characteristics

Moderate positive associations with MPI were found for having tested for HIV, own HIV status, disclosure of HIV status to partner, condom use at last sex, having talked to healthcare provider about trying to get pregnant in the future, planning to have more children, condom use to prevent HIV and STIs, importance of partner’s desire to have children, importance of healthcare provider’s advice about whether to get pregnant or not, and PMTCT knowledge. Furthermore, small negative associations with MPI were found for alcohol use.

#### 3.3.3. Partner Characteristics

Finally, moderate positive associations with MPI were found for awareness of female partner having tested for HIV and partner HIV status. Small positive associations were found with partner’s use of reasoning skills to resolve conflict, while small negative association was found for partner’s use of verbal aggression.

### 3.4. Factors Associated with Male Partner Involvement in PMTCT

The results of the multiple linear-regression analysis are shown in Table 3. Results of the unadjusted model are described. Relationship status (specifically living together with partner), awareness of female partner’s positive HIV status, female partner’s desire to have more children in the future, having talked to a healthcare provider about trying to get pregnant in the future, planning to have more children in the future, condom use to prevent HIV and STIs, and partner’s reasoning skills, were significantly and positively associated with MPI. Partner’s verbal aggression was inversely associated with MPI. Overall, this model accounted for 30.5% of the variance in MPI (*R*^2^ = 0.305).

Table 4 summarizes the final multiple linear-regression model. Participants who reported to be living together with their partner, own HIV status, awareness of female partner’s positive HIV status, female partner’s desire to have more children in the future, having talked to healthcare provider about trying to get pregnant in the future, condom use to prevent HIV and STIs, and partner’s reasoning skills were more likely to participate in the specified male involvement activities. Participants who reported partner’s verbal aggression were less likely to participate. Overall, the final model accounted for 31% of the variance in MPI (*R*^2^ = 0.310).

## 4. Discussion

This study reports on the prevalence of male partner involvement and highlights some important factors that influence male partner involvement in PMTCT programs in rural South Africa.

The results of this study indicated that 44.1% of male partners reported involvement in most or all MPI activities, that is, scores of 7 to 9. This figure is higher compared to what has been reported in similar studies [23,24]. The prevalence of MPI in this study is being compared with only those in similar studies conducted in PMTCT contexts rather than generally in maternal and infant care. In north-west Ethiopia and Addis Ababa, 20.9% and 28.1% of men had high MPI (score of 4–6) in PMTCT [23,24]. However, this figure is low compared to what has been reported from a most-recent similar study in southern Ethiopia where the level of MPI (score of 4–6) in a PMTCT program was 53% [16]. Low levels of male partner involvement in maternal and infant health and PMTCT are partly attributed to traditional gender norms prevalent in patriarchal societies, which make involvement by men in such issues a challenge [32,33,34,35,36].

In this study, 10.6% of men reported involvement in all the listed activities (see Table 2), while 3.7% reported no involvement in any of the activities. The theory of planned behavior (TPB) [37], one of the most commonly used frameworks to explain behavior, proposes that intention, the most important determinant of behavior, is determined by individuals’ attitudes (positive or negative evaluation of performing a particular behavior), subjective norms (perceived social expectations) and perceived behavioral control (self-efficacy). MPI in PMTCT programs may be greatly influenced by community held traditional gender norms (subjective norms) where most men gave limited support to their HIV-positive pregnant partners as they believe that some of the activities are “women matters”. However, some men still chose to fully support their partners regardless of these traditional gender norms (positive attitude and self-efficacy). Interventions should focus on changing men’s negative attitudes towards involvement in maternal and infant-health care in PMTCT settings. On a larger scale, there is a need to explore a shift in gender norms. For example, this can be achieved through the adaptation and implementation of evidence-based gender-transformative programs with men, which has led to positive changes in their behavior and attitudes related to maternal and infant health [1,38]. The “Men as Partners” program among South African men, for example, manipulated gender norms ascribed to traditional partner relations and challenged male attitudes and behaviors that compromised the health of women [39]. The Ekialodor safe motherhood program in Nigeria conducted group health talks to improve male attitudes and practices regarding their involvement in prenatal care [40].

The finding that male partners living together with their partners were more likely to be involved is similar to the findings by other studies [18,23]. This is much to be expected, as cohabiting men and women may have more time to communicate and harmonize their time schedules [18] to allow instrumental support to the pregnant partner. Matseke et al. [34] used the concept of social support [22] to describe MPI in antenatal care. It makes sense that male partners not living with their partners may find it difficult to support their partners. However, whereas instrumental support may be difficult to achieve among non-cohabiting partners, other forms of support may be easier to achieve than others. For example, financial support is achievable amongst both non-cohabiting and cohabiting partners, while sharing of household workload may not be achievable among non-cohabiting partners. Accompaniment to health facilities maybe easier among cohabiting partners but not necessarily unachievable among non-cohabiting partners. As such, future interventions aimed at improving MPI should focus on exploring ways to harmonize time schedules among non-cohabiting partners to enable optimal involvement among men not living with their partners.

It is an interesting finding that male partners who were aware of their female partners’ HIV-positive status were more likely to engage in MPI activities. A study in Ivory Coast showed that men played an active role in applying the advice they received when they became aware that their spouse is HIV positive and involved in the PMTCT program [6]. According to the health belief model [41], behavior is determined by threat perceptions and beliefs about the benefits of the recommended action and potential barriers for its implementation. This means that a male partner took a health-related action (i.e., involvement to support an HIV-positive pregnant partner) if he feels that by so doing, a negative health condition (i.e., infant HIV infection) can be avoided. In this case, the fact that a male partner knew his partner’s HIV positive status and therefore wanted to ensure that they give birth to a healthy baby may have served as motivation for male support. Mutual disclosure of HIV status among partners is important as it allows decision making regarding healthy antenatal and postnatal care choices. Disclosure for HIV-positive women can encourage their partners to make informed reproductive health options [42]. In this study, just over a third (67.2%) of male partners knew their partner’s HIV-positive status, while 78% did not tell their female partner about their status. Male partner involvement interventions that encourages mutual HIV testing and disclosure among both partners would thus be beneficial in this regard.

Although HIV knowledge and PMTCT-specific knowledge were generally high among male participants, it was not associated with MPI. This is not surprising, since behavioral scientists have shown that knowledge does not always directly translate into behavior change [43]. Methods for successfully conveying information, as suggested by concepts of the theories of information processing [44,45], should be considered in the planning of male partner involvement interventions.

Male partners whose partners had used reasoning skills to settle couple differences were also more likely to engage in MPI activities. This finding suggests that when having differences regarding important issues, some couples resort to discussing issues calmly, or bringing in someone to help settle things.

It is not surprising that male partners who reported that their partner had used verbal aggression to resolve conflict were less to likely to be engaged in more MPI activities. This finding is similar to the results of a study among pregnant HIV-positive women, where both psychological and physical violence had been associated with poor male partner involvement in PMTCT in South Africa [46]. It makes sense that talking about HIV-related issues may bring some discomfort among some couples leading to less sufficient discussions, leaving some issues unsaid, amounting to poor communication. Poor communication between men and their female partners has been associated with poor MPI [18]. Male partner involvement interventions that promote good communication skills among partners are essential in having satisfying violence-free HIV-related and family-planning discussions.

Men can be involved in a variety of ways during and after pregnancy, not just in a single activity. Hence, this paper utilized a nine-item composite measure of MPI in PMTCT to capture a range of prenatal and postnatal activities that men could possibly engage in. Furthermore, composite measures in future studies should be adapted in the context of the area and population of interest. For example, in Matseke et al. [34], rural South African men did not necessarily view attending antenatal visits as a form of MPI, but rather, accompaniment to the clinic that included behaviors such as holding a spot in the clinic queue. In addition, MPI was understood as giving instrumental support to female partners through financial help, helping out with physical tasks and giving emotional support.

This study highlights important information for future use by policy makers in the maternal, newborn and child health areas. However, this study has its limitations. Firstly, the results may not be generalizable to other areas and populations, as the study is limited to male partners of HIV-positive pregnant women who visited CHCs in a predominantly rural province. Secondly, the study relied on self-reporting of MPI not verified otherwise, and may be under- or over-reported. Thirdly, cross-sectional data was used and therefore causality and direction of results cannot be determined; longitudinal analysis may provide additional insight into MPI by clarifying temporal associations. Finally, this study utilized secondary data and therefore does not comprehensively investigate all factors that may be associated with MPI, and these should be included in future studies.

## 5. Conclusions

Male partner involvement in this study is low and can be improved. Future MPI interventions should focus on promoting men’s positive attitudes towards involvement in maternal and infant health care. These interventions should focus more on non-cohabiting male partners, and encourage HIV testing among men and disclosure of HIV status to female partners. Good communication skills among partners are essential in enabling conflict-free HIV-related and family-planning discussions. Interventions aimed at improving MPI in PMTCT may be more beneficial, and should thus include a range of pregnancy- and infant-related activities, rather than a single activity.

## Figures and Tables

**Table 1:** Socio-demographic, partner characteristics and behavioral characteristics of the male partners (*N* = 463).

Characteristic	*n* (%) Median (IQR)
Age	
mean	33.04 (SD = 7.24)
19–49 years	452 (97.6)
≥50 years	11 (2.4)
Relationship status	
Unmarried, living separate	207 (44.7)
Unmarried, living together	169 (36.5)
Married	87 (18.8)
Length of period in current relationship	
Less than 1 year	80 (17.5)
More than 1 year	376 (82.5)
Educational attainment	
Grade 0–11	278 (60.0)
Grade 12 or more	185 (40.0)
Employment status	
Not employed	188 (40.6)
Employed	275 (59.4)
Income (Rands)	
Less than 1000	123 (26.6)
1000 or more	340 (73.4)
Number of children	
0	110 (23.8)
1	130 (28.1)
2 or more	223 (48.2)
Tested for HIV	
No	137 (29.6)
Yes	326 (70.4)
HIV status, positive	
Yes	297 (64.1)
No	166 (35.9)
Total adherence to antiretroviral (ARV) adherence	7.00 (0.00)
Self-rated adherence	5.00 (1.00)
Disclosed HIV-status partner	
No	348 (75.2)
Yes	115 (24.8)
Partner tested for HIV	
No/don’t know	85 (18.4)
Yes	378 (81.6)
Partner HIV positive	
No/don’t know	152 (32.8)
Yes	311 (67.2)
Partner currently on ARV	
No/Do not know/HIV-Partner	173 (37.4)
Yes	290 (62.6)
Child HIV positive	
No/No Children	446 (96.3)
Yes	17 (3.7)
Condom use at last sex	
No	220 (47.5)
Yes	243 (52.5)
Alcohol use in the past month (>3 drinks)	
No	276 (59.6)
Yes	187 (40.4)
AIDS-Related Knowledge scores	
Knowledge score (range 0–12)	10.00 (3.00)
Mother-to-child transmission (PMTCT) knowledge score (range 0–6)	5.00 (3.00)
Kalichman Stigma Scale—9 items	
Score (range 0–8)	1.00 (0.00)
Partner violence (Conflict Tactics Scale)	
Reasoning subscale	4.00 (7.00)
Verbal aggression subscale	1.00 (5.00)
Mild physical aggression subscale	0.00 (0.00)
Severe physical aggression subscale	0.00 (0.00)
Family planning assessment	
Biological father	432 (93.3)
Partner‘s pregnancy unplanned	211 (45.6)
Having talked with health care provider about trying to get pregnant	125 (27.0)
Planning to have more children in future	232 (50.1)
Currently using condoms to prevent HIV or STIs	324 (70.0)
Partner‘s desire to have baby (scores 0–10)	9.50 (9.00)
Health care provider‘s advice about whether to have baby or no (scores 0–10; 0 = not important at all, 10 = very important)	1.00 (9.00)

**Table 2:** Male partner involvement in PMTCT (*N* = 463).

Characteristic	*n* (%) Median (IQR)
Attends antenatal care visits with partner	
No	317 (68.5)
Yes	146 (31.5)
Knows partner’s antenatal appointment times	
No	168 (36.3)
Yes	295 (63.7)
Discussed baby antenatal health care with partner	
No	155 (33.5)
Yes	308 (66.5)
Supports partner’s antenatal visits financially	
No	71 (15.3)
Yes	392 (84.7)
Knows what happens in the antenatal clinic	
No	233 (50.3)
Yes	230 (49.7)
Have you been asked to take an HIV test?	
No	78 (16.8)
Yes	59 (12.7)
Unknown	326 (70.4)
Has your partner discussed feeding options for the baby with you?	
No	146 (31.5)
Yes	317 (68.5)
Have you discussed the place of delivery for the baby with your partner?	
No	167 (36.1)
Yes	296 (63.9)
Have you discussed testing your baby for HIV with your partner?	
No	49 (10.6)
Yes	117 (25.3)
Unknown	297 (64.1)
Have you discussed condom use with your partner?	
No	90 (19.4)
Yes	373 (80.6)
male partner involvement (MPI) scores	
0	17 (3.7)
1	23 (5.0)
2	48 (10.4)
3	30 (6.5)
4	50 (10.8)
5	41 (8.9)
6	64 (13.8)
7	86 (18.6)
8	62 (13.4)
9	42 (9.4)
MPI (index)	
MPI score (range 0–9)	6.00 (IQR = 4.00)

**Table 3:** Results of linear-regression model predicting MPI (*N* = 454).

Characteristics	Unstandardized Coefficients		Standardized Coefficients		*p*	95.0% Confidence Interval for *B*	
	*B*	SE	*B*	*t*		Lower Bound	Upper Bound
(Constant)	1.540	0.438		3.517	0.000	0.679	2.401
Relationship status: unmarried, living together (ref = unmarried, living separate)	0.594	0.229	0.113	2.595	0.010	0.144	1.044
Relationship status: married	0.081	0.287	0.012	0.282	0.778	−0.483	0.644
Number of children	0.125	0.132	0.040	0.946	0.344	−0.135	0.385
Tested for HIV (ref = not tested)	0.263	0.260	0.047	1.010	0.313	−0.249	0.775
Own HIV status (ref = negative)	0.356	0.328	0.067	1.087	0.278	−0.288	1.001
Female partner tested for HIV (ref = partner not tested)	0.534	0.364	0.082	1.467	0.143	−0.182	1.250
Female partner HIV positive (ref = no/don’t know)	0.730	0.312	0.135	2.344	0.019	0.118	1.343
Alcohol use (3 or more drinks in a day in the past month vs. not)	−0.137	0.214	−0.027	−0.642	0.521	−0.558	0.283
Condom use at last sex (ref = no)	0.036	0.246	0.007	0.147	0.883	−0.448	0.520
Discussed future pregnancy with provider (ref = no)	0.997	0.236	0.175	4.215	<0.001	0.532	1.462
Planning to have more children in the future (ref = no)	0.427	0.208	0.084	2.049	0.041	0.017	0.837
Currently using condoms to prevent HIV transmission (ref = no)	0.854	0.259	0.154	3.296	0.001	0.345	1.364
Your partner’s desire to have a baby (or not)	0.094	0.029	0.147	3.215	0.001	0.037	0.152
Health care provider’s advice about whether you should have a baby (or not)	0.012	0.028	0.018	0.414	0.679	−0.044	0.067
PMTCT knowledge	0.061	0.061	0.042	0.995	0.320	−0.059	0.181
CTS reasoning	0.068	0.025	0.118	2.767	0.006	0.020	0.116
CTS verbal aggression	−0.043	0.019	−0.096	−2.290	0.022	−0.080	−0.006
Disclosure to partner (ref = not disclosed)	0.277	0.349	0.045	0.793	0.428	−0.409	0.963

Note: CTS = Conflict Tactics Scale. *B* = Beta coefficient. SE = Standard Error. *t* = *t*-statistic. All significant *p*-values are in bold. *R*^2^ = 0.305; *F*(18,443) = 12.24, *p* < 0.001.

**Table 4:** Results of reduced multiple linear-regression model predicting MPI (*N* = 454).

Characteristics	Unstandardized Coefficients		Standardized Coefficients		*p*	95.0% Confidence Interval for *B*	
	*B*	SE	*B*	*t*		Lower Bound	Upper Bound
(Constant)	1.906	0.317		6.010	0.000	1.283	2.529
Relationship status: unmarried, living together (ref = unmarried, living separate)	0.586	0.206	0.111	2.848	0.005	0.182	0.990
Own HIV status (ref = negative)	0.687	0.230	0.130	2.986	0.003	0.235	1.139
Female partner tested for HIV (ref = partner not tested)	0.636	0.352	0.097	1.807	0.071	−0.056	1.327
Female partner HIV positive (ref = no/don’t know)	0.780	0.300	0.145	2.597	0.010	0.190	1.371
Discussed future pregnancy with provider (ref = no)	1.062	0.230	0.186	4.626	<0.001	0.611	1.513
Planning to have more children in the future (ref = no)	0.382	0.200	0.075	1.913	0.056	−0.010	0.775
Currently using condoms to prevent HIV transmission (ref = no)	0.918	0.229	0.166	4.018	<0.001	0.469	1.367
Your partner‘s desire to have a baby (or not)	0.106	0.026	0.164	4.087	<0.001	0.055	0.156
CTS reasoning	0.066	0.024	0.114	2.746	0.006	0.019	0.112
CTS verbal aggression	−0.045	0.018	−0.101	−2.452	0.015	−0.081	−0.009

Note: CTS = Conflict Tactics Scale. *B* = Beta coefficient. SE = Standard Error. *t* = *t*-statistic. All significant *p*-values are in bold. *R*^2^ = 0.310; *F*(10,451) = 21.75, *p* < 0.001.

## Supplementary Materials

The following are available online at http://www.mdpi.com/1660-4601/14/11/1333/s1, Correlations among Key Study Variables (*N* = 463).
