# Supplementary material for: Factors Associated with Male Partner Involvement in Programs for the Prevention of Mother-to-Child Transmission of HIV in Rural South Africa
Source: Int J Environ Res Public Health. 2017 Nov 1;14(11):1333. doi: 10.3390/ijerph14111333 (PMC5707972; doi:10.3390/ijerph14111333)
Supplement: Supplementary file 1 [file ijerph-14-01333-s001.pdf]

Appendix A: Correlations among Key Study Variables (N= 463)

	MI	1	2	3	4	5	6	7	8	9	10	11	12	13	14	15	16	17	18	19	20	21	22	23	24	25	26	27	28	29	30
MI	1																														
1. AGE	-0.022	1																													
2. JOB	-0.013	0.015	1																												
3. ED	0.09	.155**	.112*	1																											
4. INC	0.054	-0.037	.437**	0.086	1																										
5. RSa	.184**	0.03	-0.004	-0.051	0.01	1																									
6. RSb	0.044	-0.07	0.06	-0.032	-0.01	-.365**	1																								
7. RL	0.058	-0.049	0.044	-0.031	-0.029	-.354**	1.000**	1																							
8. #CH	.122**	-.145**	-0.066	-.211**	0.055	0.07	.196**	.191**	1																						
9. MTEST	.233**	0.023	-0.005	0.084	0.018	0.089	-0.064	-0.077	0.014	1																					
10. MHIV	.266**	-0.061	-0.027	-0.03	-0.059	0.088	0.032	0.019	.169**	.485**	1																				
11. TEST	.326**	0.036	-.115*	0.014	0.03	.093*	0	0.008	0.074	.279**	.168**	1																			
12. PS	.358**	-0.018	-.093*	-0.029	0.046	.119*	0.054	0.073	.091*	.242**	.321**	.678**	1																		
13. CS	-0.035	-0.045	0.068	-0.052	-0.029	0.019	0.053	0.037	.139**	0.026	.117*	.093*	.112*	1																	
14. ALC	-.129**	-0.016	0.025	0.028	-0.081	-0.057	-.092*	-.097*	-.112*	-0.045	-.101*	-0.019	-.118*	-0.044	1																
15. SADH	0.025	-0.124	0.09	0.12	0.159	0.051	0.059	0.035	-0.057	.c	.c	-0.149	-0.091	-0.166	- .296**	1															
16. ADH	0.036	-0.093	0.01	.198*	.291**	0.058	0.021	0.014	0.171	.c	.c	0.125	0.159	-0.02	-0.123	.312**	1														
17. CU	.289**	0.05	-0.081	0.085	0.029	.155**	0.048	0.057	.110*	.208**	.215**	.230**	.293**	0.025	- .195**	0.021	0	1													
18. BIO	0.064	-0.042	0.006	0.013	0.059	-0.012	0.062	0.056	0.08	.129**	0.038	.118*	.107*	0.006	-0.008	.230*	0.152	.091*	1												
19. PU	-0.007	0.062	-0.002	-0.084	-0.025	0.056	-0.031	-0.03	-0.019	.099*	0.027	-0.041	-0.083	0.028	0.068	0.078	0.056	-0.018	-0.012	1											
20. FP	.290**	0.063	0.016	0.053	-0.089	0.064	.106*	.099*	.093*	.160**	.215**	0.062	0.083	0.062	-0.064	-0.094	-0.078	.189**	-0.032	0.039	1										
21. MC	.103*	.100*	0.025	0.063	0.071	-0.015	0.004	0.019	-.237**	0.035	-.110*	0.085	0.011	-0.035	0.038	-.247**	0.027	-0.024	-0.043	0.022	0.072	1									
22. CU HIV	.328**	0.083	-0.078	.111*	-0.063	0.046	0.074	0.086	0.063	.215**	.185**	.249**	.214**	-0.048	- .133**	-0.041	0.062	.519**	0.088	0.063	.165**	0.025	1								
23. PART	.237**	0.01	-0.042	.106*	0.088	0.055	0.053	0.049	0.081	-0.084	-.108*	.140**	.141**	-0.049	- .143**	0.013	0.182	.171**	0.051	-.095*	0.036	0.083	.116*	1							
24. PROV	.107*	-0.013	-0.019	.123**	-0.024	-0.046	0.071	0.062	0.068	-0.036	-0.031	0.016	-0.047	0.004	0.091	-0.117	0.119	0.084	-0.025	0.06	.165**	.103*	0.051	.378**	1						
25. PMTCT KN	.162**	0.028	0.014	.128**	.097*	0.074	0.008	0.022	0.077	.092*	.126**	.181**	.217**	0.001	- .153**	0.179	0.1	.135**	.147**	-.144**	0.045	-0.024	0.08	.170**	-0.075	1					
26. HIV KN	0.091	0.04	0.025	.250**	.164**	0.006	-0.005	0.007	0.04	.133**	0.058	.192**	.152**	0.003	-0.089	0.067	.202*	0.076	0.085	-.173**	-.108*	-0.066	0.082	.113*	-0.083	.512**	1				
27. REAS	.173**	-0.032	-0.041	0.018	-.098*	0.029	.107*	.102*	0.086	.141**	.176**	0.088	0.057	-0.001	0.081	0.137	-0.045	0.029	-0.082	0.07	.108*	0.012	.126**	0.013	.101*	-.131**	-0.078	1			
28. VERAG	-.127**	-0.049	0.059	0.016	-0.048	-0.029	-0.012	-0.086	-0.015	0.041	0.02	-0.071	-.118*	0.054	.145**	-0.03	0.001	-.169**	-.151**	0.039	0.011	-0.027	-.096*	-.110*	0.066	-.154**	-.114*	.284**	1		
29. DSCP	.244**	-0.055	-0.071	0.02	-0.019	0.034	0.027	0.034	.105*	.342**	.707**	.250**	.369**	.147**	-0.04	-0.089	0.003	.157**	0.037	0.023	.162**	-0.08	.163**	-0.077	0.005	.140**	0.09	.091*	-0.013	1	
30. PHAG	-0.071	-0.055	0.084	0.049	-0.083	0.008	-0.007	-0.083	-.101*	-0.009	-0.024	-0.052	-0.072	0.064	0.068	-0.026	0.029	-.144**	-0.07	0.019	0.017	0.023	-0.072	-.136**	-0.028	-.136**	-.159**	.146**	.643**	-0.033	1
31. STIG	0.057	-0.043	-0.021	-.269**	-.180**	0.049	-0.072	-0.064	0.054	-0.065	0.042	-0.066	-0.059	0.029	-0.039	-.212*	-0.098	0.049	-.095*	.114*	.116*	0.082	0.066	-0.072	0.004	-.116*	-.262**	0.001	0.083	-0.024	0.091

Note: ** Correlation is significant at the 0.01 level (2-tailed). * Correlation is significant at the 0.05 level (2-tailed). c Cannot be computed because at least one of the variables is constant.

MI = Male Involvement. 1. AGE = Age. 2. JOB = Employment. 3. ED = Education. 4. INC = Monthly Income. 5.RSa = Relationship Status (Unmarried & living together vs. separately). 6. RSb = Relationship Status (married). 7. RL = Relationship Length. 8. #CH = Number of Children. 9. MTEST = Male participant HIV test.

10. MHIV = Male participant HIV Status. 11. TEST = Spouse Tested for HIV. 12. PS = Partner HIV Status.

13. CS = Child Status. 14. ALC = Alcohol Use. 15. SADH = Self-Rated Adherence. 16. ADH = Adherence. 17. CU = Condom Use. 18. BIO = Biological Father of Baby. 19. UP = Unplanned Pregnancy. 20. FP = Discussion with Provider about Future Pregnancy. 21. MC = Planning to Have More Children.

22. CU HIV = Condom Use to Prevent. 23. PART = Partner Importance. 24. PROV = Provider Importance. 25. PMTCT KN = PMTCT Knowledge. 26. HIV KN = HIV Knowledge. 27. REAS = CTS Reasoning. 28. VERAG = CTS Verbal Aggression. 29. DSCP = Disclosure of HIV Status to Partner. 30. PHAG = CTS Physical Aggression. 31. STIG = Stigma.
